# Are Salivary and Plasma Levels of Toll-Like Receptors 2 and 4 Elevated in Subjects With Chronic Periodontitis?: A Systematic Review and Meta-Analysis

**DOI:** 10.1155/ijin/7405066

**Published:** 2025-05-06

**Authors:** Mario Alberto Alarcón-Sánchez, Ruth Rodríguez-Montaño, Sarah Monserrat Lomelí-Martínez, Cristina Hermila Martínez-Bugarin, Seyed Ali Mosaddad, Artak Heboyan

**Affiliations:** ^1^Molecular Biology in Medicine Program, University Center of Health Sciences, University of Guadalajara (CUCS-UdeG), Guadalajara 44340, Jalisco, Mexico; ^2^Institute of Research in Dentistry, Department of Integral Dental Clinics, University Center of Health Sciences, University of Guadalajara (CUCS-UdeG), Guadalajara 44340, Jalisco, Mexico; ^3^Department of Health and Illness As an Individual and Collective Process, University Center of Tlajomulco, University of Guadalajara (CUTLAJO-UdeG), Tlajomulco de Zuñiga 45641, Jalisco, Mexico; ^4^Department of Medical and Life Sciences, La Ciénega University Center, University of Guadalajara (CUCIÉNEGA-UdeG), Ocotlán 47820, Jalisco, Mexico; ^5^Department of Research Analytics, Saveetha Dental College and Hospitals, Saveetha Institute of Medical and Technical Sciences, Saveetha University, Chennai, India; ^6^Department of Conservative Dentistry and Bucofacial Prosthesis, Faculty of Odontology, Complutense University of Madrid, Madrid, Spain; ^7^Department of Prosthodontics, School of Dentistry, Shiraz University of Medical Sciences, Shiraz, Iran; ^8^Department of Prosthodontics, Faculty of Stomatology, Yerevan State Medical University After Mkhitar Heratsi, Str. Koryun 2, Yerevan 0025, Armenia

**Keywords:** biomarkers, periodontitis, plasma, saliva, TLR2, TLR4

## Abstract

Toll-like receptors (TLR2 and TLR4) are crucial in the detection of pathogen-associated molecular patterns (PAMPs) during periodontitis, resulting in exacerbated production of proinflammatory cytokines and ultimately tissue damage and bone loss associated with this periodontal disease. This systematic review and meta-analysis aimed to systematically analyze and quantify the differences between TLR2 and TLR4 levels in the saliva and plasma of individuals with chronic periodontitis (CP) and systemically and periodontally healthy subjects (SPHS). The databases consulted were Scopus, Web of Science, and PubMed from 2011 to 2024 to locate cross-sectional studies that measured TLR2 and TLR4 levels. Studies selected were human research articles published in English, evaluating these biomarkers through ELISA. Data were extracted, and the quality of studies was appraised using the Joanna Briggs Institute (JBI) tool for observational studies. Meta-analyses were executed using STATA V.15 (StataCorp LP, College Station, Texas) employing fixed or random-effects models based on the degree of heterogeneity using I^2^ statistics. Out of 404 articles found, four studies were included for both qualitative and quantitative synthesis. We found an increase in salivary TLR4 levels in subjects with CP compared with SPHS (SMD = 265.217 (95% confidence interval (CI) = 109.311–421.122); *p*=0.001). As well as an increase in plasma levels of TLR4 in subjects with CP compared with SPHS (SMD = 2.93 (95% CI = 1.57–4.29); *p*=0.001). TLR4 concentrations in saliva and plasma of subjects with CP were higher than those observed in the healthy population. However, further validation in larger prospective studies is needed before clinical implementation.

## 1. Introduction

Periodontitis is among the most prevalent chronic immunoinflammatory disorders in the world (64%) [1]. In fact, it is considered the sixth most common osteolytic disease after rheumatoid arthritis [2]. Clinically, individuals with this condition present with gingival inflammation with bleeding and suppuration, as well as tooth mobility and bone loss [3]. Untreated periodontitis leads to tooth loss and partial edentulism [4]. Therefore, early diagnosis and timely intervention are important to prevent these secondary events [5]. In general, the most common cause of its occurrence is bacterial aggression produced by an increased prevalence of orange and red complex periodontopathogens, together with the presence of a dysregulated immune response in a genetically susceptible host [6].

Upon bacterial aggression, pathogen-associated molecular patterns (PAMPs) such as lipopolysaccharide (LPS) from Gram-negative anaerobes [7] can stimulate host cells (keratinocytes, gingival fibroblasts, neutrophils, and macrophages) through their interaction and subsequent binding to different pattern recognition receptors such as Toll-like receptors (TLRs); TLR2 and TLR4 [8] and consequently activate the nuclear factor kappa B (NF-κB) pathway that upregulates the expression of genes involved in the inflammatory and destructive process (cytokines, chemokines, and matrix metalloproteases (MMPs), which lead to the translation of proteins such as tumor necrosis factor-alpha (TNF-α), interleukin 6 (IL-6), interleukin 1 beta (IL-1β), interleukin 8 (IL-8), MMP-2, MMP-8, MMP-9, and MMP-13, which favors the degradation of the extracellular matrix of the gingival tissue and properly to bone resorption, two events highly characteristic of the disease [9].

Clinical evaluation (dentoalveolar radiographic examination and periodontal probing) continues to be the best diagnostic method to assess the periodontal condition of individuals [10]. However, as part of biochemical and molecular advances, the need arises to improve these tests and implement the use of biomarkers that have the ability to distinguish between a state of periodontal health and, as such, reflect the destructive process [11]. Thus, plasma and saliva are biofluids constituted by a wide variety of biomolecules, which can give us a clear idea about the systemic and oral condition of subjects with periodontitis [12].

Significant changes in plasma and salivary TLR2 and TLR4 levels have been reported in individuals with chronic periodontitis (CP); however, to the authors' knowledge, there is no systematic review analyzing these results.

Therefore, the goal of the present systematic review and meta-analysis was to investigate the changes occurring in the protein levels of TLR2 and TLR4 in the saliva and plasma of individuals with CP compared with systemically and periodontally healthy subjects (SPHSs).

## 2. Materials and Methods

The research conformed to PRISMA guidelines. The study protocol was submitted in OSF, accessible through https://doi.org/10.17605/OSF.IO/3YWCN.

### 2.1. Research Question

Are salivary and plasma levels of TLR2 and TLR4 elevated in subjects with CP compared to SPHS?

### 2.2. PECO Algorithm

1. P (Population): subjects with CP.
2. E (Exposure): TLR2 and TLR4 levels in saliva and plasma.
3. C (Control): periodontal healthy subjects.
4. O (Outcomes): differences/changes in TLR2 and TLR4 levels in saliva and plasma.

### 2.3. Eligibility Criteria

Observational cross-sectional clinical studies evaluating salivary and plasma levels of TLR2 and TLR4 by enzyme-linked immunosorbent assay (ELISA) were included. Regarding the study population included, participants had to be of both sexes and over 18 years of age. Individuals with CP had to have a probing depth (PD) and clinical insertion loss (CAL) greater than or equal to 4 mm. In addition, they should have no pre-existing comorbidities and no history of taking medications such as antibiotics or anti-inflammatory drugs, as well as periodontal therapy in the last months prior to the study. Consideration was given only to studies published in English in peer-reviewed journals. Exclusion criteria included individuals with any systemic disease, those who had undergone periodontal treatment within the last 6 months, smokers, alcohol consumers, pregnant women, individuals receiving orthodontic treatment, and those on antibiotics and/or immunosuppressive treatment. Furthermore, studies involving cell lines and animal models, as well as randomized clinical trials, systematic, comprehensive or narrative reviews, editorials, and letters to the editor, were also excluded.

### 2.4. Strategy and Study Selection

The following three electronic databases: Scopus, Web of Science, and PubMed, were consulted from September 15, 2011, to January 15, 2024. The search strategy is summarized in Table 1. Keyword included “Toll-Like Receptor 2,” “Toll-Like Receptor 4,” “Saliva,” “Plasma,” “Periodontitis,” and “Chronic Periodontitis.” The manual search was carried out in different journals related to periodontics, microbiology, and oral immunology. Subsequently, 2 researchers (M.A.A.-S. and R.R.-M) reviewed the titles and abstracts resulting from the literature search based on predefined eligibility criteria. Articles with abstracts relevant to the topic were selected for full-text review, while those deemed irrelevant were discarded. Any disagreements were settled through group discussion.

### 2.5. Data Collection

The data extraction process was performed independently by two authors (M.A.A.-S. and A.H.). Initially, data were extracted and recorded on a server sheet using Excel. The extracted data included name of first author, year, countries, title, goal, design of the study, approval of the ethical committee, name of the journal, including and excluding criteria, genders, ages, the number of cases (individuals with CP) and controls (SPHS), the size of the study population, periodontal criteria and periodontal clinical parameters PD, CAL, plaque index (PI), bleeding on probing (BOP), and radiographic bone loss (RBL). In addition, the collection of biological samples (saliva and plasma), type of biomarker, immunoassay technique used, mean ± standard deviation (SD)/median and interquartile range, *p* value, and primary results and conclusions were documented.

### 2.6. Quality Assessment of Included Studies

Two evaluators (S.M.L.-M. and C.H.M.-B.) independently appraised the quality of the included cross-sectional studies using the Joanna Briggs Institute (JBI) tool [13]. Responses were categorized as “Yes,” “No,” “Unclear,” or “Not applicable.” The studies were then ranked based on their quality into three categories: low quality for studies scoring up to 49%, moderate quality for those scoring between 50% and 69%, and high quality for studies scoring above 70%.

### 2.7. Statistical Analysis

The standardized mean difference (SMD) in TLR2 and TLR4 levels were compared between study groups (CP vs. SPHS) using a fixed or random effects model depending on the heterogeneity detected. Heterogeneity was performed with was the Chi^2^ test and the I^2^ statistic, taking low (25%–49%), moderate (50%–75%), and high (75%–100%). A *p* value of less than 0.05 was sufficient to be significant. Forest plots were created to display estimates with a 95% confidence interval (CI), and publication bias was evaluated using funnel plots and Egger's linear regression. Meta-analyses were performed with STATA V.15 (StataCorp LP, College Station, Texas).

## 3. Results

### 3.1. Study Selection

**Figure 1:**
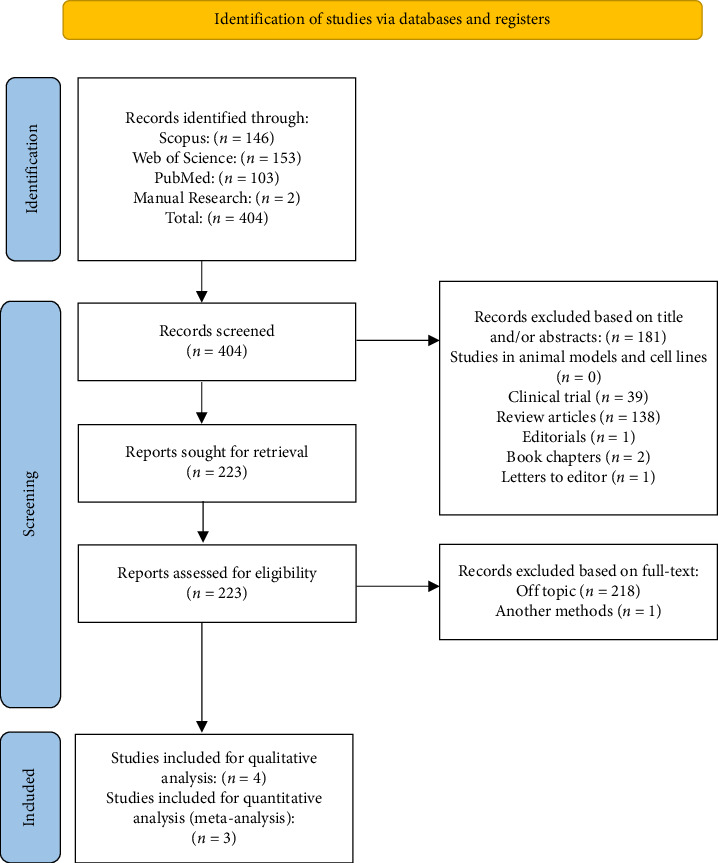
PRISMA flow diagram of the study selection process. PRISMA: preferred reporting items for systematic and meta-analyses.

Initially, 402 articles were identified within the searched databases. Through manual searching, an additional 2 articles were discovered, totaling 404 articles. During the screening phase, with the application of eligibility criteria, 181 articles were excluded for the following reasons: clinical trials (*n* = 39), reviews (*n* = 138), editorials (*n* = 1), book chapters (*n* = 2), and letters to the editor (*n* = 1), resulting in 223 potentially relevant articles. Upon full-text analysis, 218 articles were discarded as they did not pertain to the topic of interest, and one article was omitted due to a differing methodology, thus not qualifying for the meta-analysis. Consequently, 4 articles were selected for qualitative analysis, and among these, 3 were subjected to quantitative analysis in this review. The process of study selection is depicted in Figure 1.

### 3.2. Sociodemographic, Clinical, and Immunologic Characteristics of Included Studies

This study reviewed a total of 4 articles with a cross-sectional design [14–17]. The earliest study dates back to 2011 [17], while the most recent is from 2024 [14]. These studies were published across three different countries [14–17]. Half of the studies (50%) were conducted in Turkey [14, 17], and the remaining studies (25% each) were carried out in the USA [15] and India [16]. Table 1 further details the titles and objectives of these studies, as well as the journals in which they were published.

All projects received approval from the ethics committees of the respective institutions [14–17]. The case group inclusion criteria were subjects with CP presenting a PD of > 4 mm and CAL of > 4 mm [14–17]. The most common exclusion criteria included subjects with systemic diseases (100%) and those undergoing treatment with antibiotics and/or anti-inflammatory drugs (100%) [14–17]. The total number of participants in the included studies was 173, with 102 in the case group (individuals with CP) and 71 in the control group (SPHS). The participants' ages ranged from 30 to 65 years, with an average age of 39.24 years. Women constituted 47.4% of the study population, while men made up 52.6% [14–17] (Table 2).

### 3.3. Quality of Studies According to the JBI Tool

**Figure 2:**
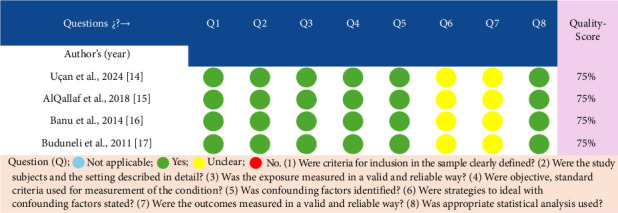
Quality of the included studies assessed through the JBI items.

All studies considered for this review were of moderate quality [14–17] (Figure 2).

### 3.4. Meta-Analysis: Comparison of Salivary TLR2 and TLR4 Levels in Subjects With CP and SPHS

**Figure 3:**
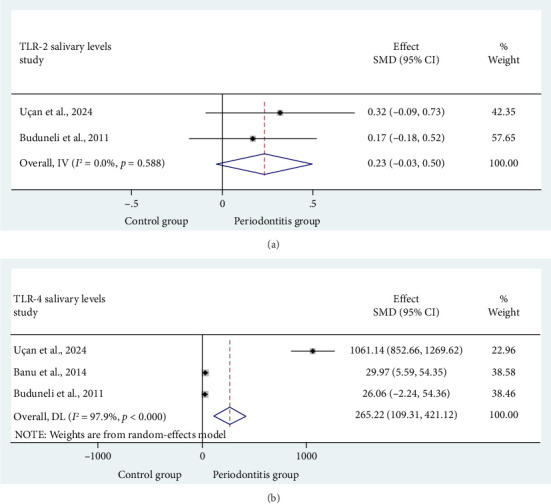
Forest plot comparing the TLR2 and TLR4 levels in saliva of (a-b) control group vs. periodontitis group.

Figure 3(a), illustrates that two articles [14, 17] assessed the salivary TLR2 level differences between CP patients (*n* = 42) and SPHS (*n* = 41). The results indicated an increase in salivary TLR2 levels in comparison with the healthy population (SMD = 0.234 (95% CI = −0.03–0.50); *p*=0.08), although this was not statistically significant. With low study heterogeneity (I^2^ = 0.0%, *p*=0.588), a fixed-effects model was applied to combine the findings.

**Figure 4:**
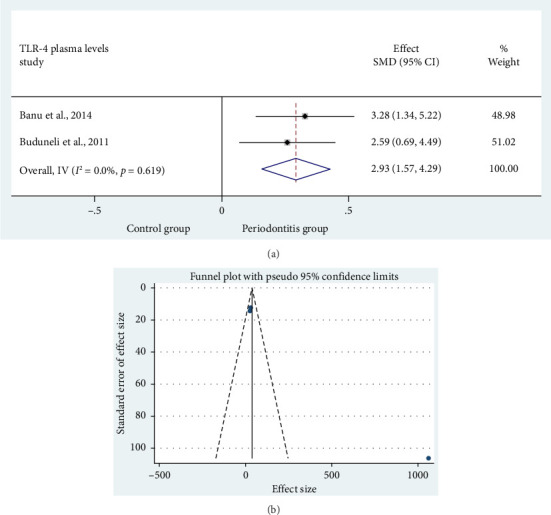
Forest plot comparing the TLR4 levels in plasma of (a) Control group vs. periodontitis group. (b) Funnel plot to check the publication bias.

Figure 3(b) shows that three articles [14, 16, 17] examined the difference in salivary TLR4 levels between CP patients (*n* = 82) and SPHS (*n* = 61). There was a notable increase in salivary TLR4 levels when compared with the healthy population (SMD = 265.21 (95% CI = 109.31–421.12); *p*=0.001). Due to high study heterogeneity (I^2^ = 97.9%, *p*=0.001), a random-effects model was utilized for result synthesis. The funnel plot indicated asymmetry and the potential for publication bias. However, Egger's test (*t* = 7.04, *p*=0.090) revealed no significant bias (refer to Figure 4(b)).

### 3.5. Meta-Analysis: Comparison of Plasma TLR4 Levels in Subjects With CP and SPHS

In Figure 4(a), two studies [16, 17] assessed the differences in plasma TLR4 levels between CP patients (*n* = 62) and SPHS (*n* = 41). There was a notable rise in plasma TLR4 levels in comparison with the healthy population (SMD = 2.93 (95%CI 1.57–4.29); *p*=0.001). Given the low heterogeneity of the study (*I*^2^ = 0.0%, *p*=0.619), a fixed-effect model was employed to aggregate the findings (Table 3).

## 4. Discussion

TLRs (TLR1–TLR11) are receptors crucial for the innate and adaptive immune responses, recognizing various microbial products and triggering inflammation [18, 19]. Notably, the periodontal microbiota role in starting and advancing periodontitis via the TLR2/4-NF-κB signaling pathway has been highlighted [20].

**Figure 5:**
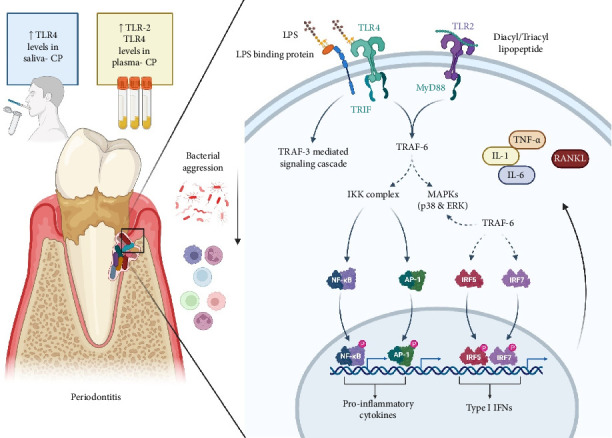
TLR signaling pathway. Particularly TLR2/4-NF-κB signaling pathway is important for the production of proinflammatory cytokines that up-regulate receptor activator of nuclear factor kappa B ligand (RANKL) leading to osteoclastogenesis. Created with https://www.BioRender.com.

Studies have indicated that bacterial species such as *Prevotella intermedia*, *Fusobacterium nucleatum*, *Aggregatibacter actinomycetemcomitans*, and *Porphyromonas gingivalis* elevate TLR2 and TLR4 gene expression and cytokine production (IL-1β, IL-6, IL-8, and IL-10) in human periodontal ligament cells [21]. In the case of bridging colonizers, *F. nucleatum* strains (*F. nucleatum* ssp. *vincentii* and ssp. *nucleatum*) have been found to significantly boost apoptosis and NF-κB mRNA expression in neutrophils, along with increased TNF-α and IL-8 levels compared to unexposed cell lines [22]. Regarding late colonizers, the initial inflammatory response to *P. gingivalis* in naïve macrophages is MyD88-dependent, necessitating collaborative TLR2 and TLR4 signaling, which then leads to cytokine production such as TNF, contributing to inflammatory bone loss [23]. Thus, the binding of periodontopathogen-derived PAMPs to TLRs in gingival tissue cells is a critical step for producing proinflammatory mediators involved in bone resorption, as seen in periodontitis (Figure 5).

The significant role of TLRs in periodontitis was underscored through a systematic literature review and subsequent meta-analysis, which assessed the association between plasma and salivary levels of TLR2 and TLR4 in individuals with CP versus SPHS. Biomarkers, typically measured in blood (plasma/serum), tissue, or saliva samples, offer crucial insights into the condition of periodontal lesions, such as gingivitis and periodontitis, by revealing key aspects such as severity, inflammation extent, and immune response in a clinical setting [24].

Uçan Yarkaç et al. reported an increase in TLR2 and TLR4 levels correlating with the severity of periodontal disease (*p* < 0.01). Both receptors positively correlated with clinical parameters: PD, PI, GI, and CAL (*p* < 0.01). ROC curve analysis indicated that TLR4 had higher specificity and sensitivity than TLR2 in diagnosing periodontal disease [14]. AlQallaf et al. [15] evaluated the soluble forms of these receptors (sTLR2 and sTLR4). They observed higher sTLR2 mRNA expression in subjects with gingivitis compared with those healthy or with periodontitis, although not statistically significant. Conversely, sTLR4 mRNA expression was elevated in subjects with periodontitis, followed by those with gingivitis and the control group (*p* < 0.05). This study also noted inverse receptor levels, with both being higher in subjects with gingivitis compared with those with periodontitis (*p* < 0.05) and the control group (*p* > 0.05). In addition, sTLR2 inversely correlated with CAL, while sTLR4 showed a positive correlation with CAL in the gingivitis group. The authors suggested that salivary assessments of sTLR2 and sTLR4 could serve as clinically relevant markers for the progression from gingivitis to periodontitis. Banu et al. found increased plasma and salivary levels of TLR4 and IL-18 in subjects with CP compared to healthy individuals (*p* < 0.05), suggesting that high plasma levels of these markers might play a significant role in the production of proinflammatory cytokines by peripheral blood mononuclear cells and indicate a systemic immune response to periodontopathogens, potentially explaining their association with other systemic diseases. Finally, these findings agree with the study of Buduneli et al. who demonstrated increased salivary levels of TLR4 but not TLR2 and both receptors in the plasma of subjects with CP compared with healthy individuals (*p* < 0.05). In addition, plasma TLR4 levels were positively correlated with some clinical indicators such as PI % and BOP % (*p* < 0.05) [17].

The findings published in the literature mark the beginning of the investigation of TLRs as potential markers of periodontal disease. The results are positive and promising; therefore, future research with a larger study population and where other members of this receptor family are analyzed in different biofluids is recommended.

The study of TLR2 and TLR4 receptors in saliva and plasma improves the understanding of the disease by helping to understand how the activation of these receptors influences inflammation and progression of periodontitis. Furthermore, it opens the possibility that they could be studied as biomarkers for early diagnosis and/or to predict the severity or progression of periodontitis. In this way, there would be the possibility of developing immunomodulatory therapies to reduce inflammation and prevent tissue damage.

The study encountered several limitations despite attempts to include studies with similar characteristics. Significant heterogeneity could have been influenced by several factors such as the inclusion of only four studies with a cross-sectional design and small sample size. Significant heterogeneity could have been influenced by several factors such as the inclusion of only four studies with a cross-sectional design and small sample size, as well as differences in age, sex, hormonal profiles, microbial composition, and methods and criteria for collection and selection of biological samples (saliva and plasma). It is also critical to recognize that current research has not established the levels of other TLRs in plasma, serum, saliva, or gingival crevicular fluid in individuals with different stages and grades of periodontitis, which calls for caution in interpreting the results. In addition, measuring changes in TLR2 and TLR4 levels before and after treatment may help to assess the effectiveness of periodontal treatments. Finally, these receptors could link periodontitis to systemic diseases since they are involved in generalized inflammation. Consequently, further high-quality studies are needed to corroborate these findings.

## 5. Conclusions

Despite these limitations, this work provides evidence for increased TLR4 concentrations in saliva and plasma of subjects with CP compared with SPHS. However, further validation in larger prospective studies is needed before clinical implementation.

## Figures and Tables

**Table 1:** The full search strategy used in Scopus, Web of Science, and PubMed.

Database	Search strategy	Results
Scopus	(((((((((“Toll-Like Receptor 2” [Mesh]) AND “Toll-Like Receptor 4” [Mesh]) OR “Receptors, Pattern Recognition” [Mesh]) AND “Biomarkers” [Mesh]) AND “Salivary Proteins and Peptides” [Mesh]) OR “Saliva” [Mesh]) AND “Periodontal Diseases” [Mesh]) OR “Periodontitis” [Mesh]) OR “Chronic Periodontitis” [Mesh]) OR “Periodontics” [Mesh]	146
Web of Science	(((((((((“Toll-Like Receptor 2” [Mesh]) AND “Toll-Like Receptor 4” [Mesh]) OR “Receptors, Pattern Recognition” [Mesh]) AND “Biomarkers” [Mesh]) AND “Salivary Proteins and Peptides” [Mesh]) OR “Saliva” [Mesh]) AND “Periodontal Diseases” [Mesh]) OR “Periodontitis” [Mesh]) OR “Chronic Periodontitis” [Mesh]) OR “Periodontics” [Mesh]	153
PubMed	((((“Toll-Like Receptor 2” [Mesh]) OR “Toll-Like Receptor 4” [Mesh]) AND “Saliva” [Mesh]) OR “Plasma” [Mesh]) AND “Periodontitis” [Mesh]	103

**Table 2:** Demographic, clinical, and immunological features of the included study.

Author/year/reference								
Study features	Uçan et al. 2024 [14]		AlQallaf et al. 2018 [15]		Banu et al. 2014 [16]		Buduneli et al. 2011 [17]	
Country	Turkey		USA		India		Turkey	
Title	Associations between immune-inflammatory markers, age, and periodontal status: a cross-sectional study		Differential profiles of soluble and cellular toll like receptor (TLR)-2 and -4 in chronic periodontitis		Correlation of TLR-4, IL-18, transaminases, and uric acid in the patients with chronic periodontitis and healthy adults		Salivary and plasma levels of Toll-like receptor 2 and Toll-like receptor 4 in chronic periodontitis	
Objective	To investigate age-related differences in salivary immune marker (IL-17, IL-18, TNF-α, TLR-2, and TLR-4) levels comparing young and elderly individuals		To detect potential correlations between the soluble and epithelial cell associated expression of TLR-2 and TLR-4 in CP and possible use as biomarkers to assess the status and progression of the periodontal diseases		To examine the level of TLR-4, IL-18, and uric acid in the plasma and saliva of healthy individuals and patients with periodontitis		To investigate the salivary and plasma concentrations of TLR-2 and TLR-4 in patients with CP compared to subjects who are periodontally healthy	
Design	Cross-sectional		Cross-sectional		Cross-sectional		Cross-sectional	
Ethics Committee Approval	Yes		Yes		Yes		Yes	
Journal	Odontology		PLOS ONE		Journal of Periodontology		Journal of Periodontology	
Inclusion Criteria	Subjects with CP		Subjects with CP		Subjects with CP		Subjects with CP	
Exclusion Criteria	Systemic diseases, pregnancy, antibiotics/inflammatory drugs, smokers, alcohol use, periodontal treatment in the previous 6 months		Systemic diseases, antibiotics/inflammatory drugs, smokers, periodontal treatment in the previous 6 months		Systemic diseases, infections, pregnancy, antibiotics/inflammatory drugs, smokers		Systemic diseases, infections, pregnancy, antibiotics/inflammatory drugs, periodontal treatment in the previous 6 months	
Gender F^e^/M^a^	21/19		8/18		37/23		16/27	
Age (M/R)	32.82		40.22		40–65		44.5	
n (HC/CP)	20/20		10/20		20/40		21/22	
n Total	40		30		60		43	
Periodontal criteria	≥ 4 mm PD, ≥ 4 mm CAL, 80% of BOP and RBL evidence		> 30% sites having > 4 mm CAL		≥ 5 mm PD, ≥ 4 mm CAL, 80% of BOP and RBL evidence		≥ 5 mm PD, ≥ 4 mm CAL, ≥ 50% of RBL	

Clinical parameters	SPHS	CP	SPHS	CP	SPHS	CP	SPHS	CP
PD (mm)	1.82 ± 0.03	4.34 ± 0.25	0.9 ± 0.3	3.55 ± 0.88	2.28 ± 0.13	5.55 ± 0.29	1.6 ± 0.32	3.91 ± 0.98
CAL (mm)	0	4.53 ± 0.53	1.1 ± 0.3	3.97 ± 1.01	DNS	DNS	0	5.4 ± 1.59
BOP (%)	DNS	DNS	1.9 ± 0.06	38.1 ± 24.15	DNS	DNS	5 ± 3	72 ± 30
PI (%)	1.14 ± 0.08	1.51 ± 0.12	13 ± 1.3	63.28 ± 22.49	0.31 ± 0.10	1.97 ± 0.17	18 ± 16	95 ± 44
Plasma	No		No		Yes		Yes	
Saliva Type	Unstimulated		Unstimulated		NR		Stimulated	
collection Technique	DNS/5 min		Passive drooling/10 min		Expectoration/5 min		Expectoration	
Marker Type	TLR2 TLR4		TLR2 TLR4		TLR4		TLR2 TLR4	
Protein evaluation method	ELISA (Elabscience Biotechnology Co. Ltd)		ELISA (R&D systems)		ELISA (EIAab)		ELISA (Uscn Life science)	
Value CG	Saliva: 0.21 ± 0.00 ng/mL Saliva: 106.37 ± 20.15 pg/mL		NR		Saliva: 12.44 ± 2.29 ng/mL Plasma: 0.99 ± 0.25 ng/mL		Saliva: 0.18 ± 0.47 ng/mL Saliva: 14.44 ± 16.55 ng/mL Plasma: 4.01 ± 8.80 ng/mL Plasma: 0.97 ± 1.20 ng/mL	
Value EG	Saliva: 0.32 ± 0.04 ng/mL Saliva: 1061.14 ± 112.69 pg/mL		NR		Saliva: 29.97 ± 4.96 ng/mL Plasma: 3.28 ± 0.71 ng/mL		Saliva: 0.17 ± 0.25 ng/mL Saliva: 26.06 ± 31.84 ng/mL Plasma: 11.55 ± 32.69 ng/mL Plasma: 2.59 ± 3.82 ng/mL	
*p*-value	< 0.001		< 0.05		< 0.001		< 0.05	
Main results	↑ TLR-2 and TLR-4 salivary levels in group with CP compared to CG		↓TLR-4 salivary levels in group with CP compared to CG		↑ TLR-4 levels in group with CP compared to CG		↑ TLR-4 levels in group with CP compared to CG	

*Note:* Fe, female; Ma, male; M, mean; R, range; hPDLF, human periodontal ligament fibroblasts.

Abbreviations: BOP, bleeding on probing; CAL, clinical attachment level; CG, control group; CP, chronic periodontitis; DNS, data not shown; EG, exposure group; NF-κB, nuclear factor kappa B; NR, not reported; PD, probing deep; PI, Plaque Index; RBL, radiographic bone loss; SPHS, systemically periodontal healthy subjects; TLR-2, toll-like receptor 2; TLR-4, toll-like receptor 4.

**Table 3:** Summary of the meta-analysis outcomes from the selected studies.

Groups	Studies	Test of comparison				Heterogeneity		
		SMD (95% CI)	*p* value	Model	*Z*	Chi-square	*p* value	*I* square (%)
TLR-2 (Saliva) CP vs. CG	2 [14, 17]	0.23 (−0.03–0.50)	0.08	Fixed	1.709	0.29	0.58	0.0
TLR-4 (Saliva) CP vs. CG	3 [14, 16, 17]	265.21 (109.31–421.12)	0.001	Random	3.334	93.59	0.001	97.9
TLR-4 (Plasma) CP vs. CG	2 [16, 17]	2.9 (1.57–4.29)	0.001	Fixed	4.226	0.25	0.61	0.0

Abbreviations: CG, control group; CI, confidence interval; CP, chronic periodontitis; SMD, standardized mean difference; TLR-2, toll-like receptor 2; TLR-4, toll-like receptor 4.

## Data Availability

The data supporting this study's findings will be made available from the corresponding author upon reasonable request.

## Nomenclature

PRISMA: Preferred Reporting Items for Systematic Reviews and Meta-Analyses
OSF: Open Science Framework
TNF-α: Tumor necrosis factor alpha
IL-6: Interleukin 6
IL-1β: Interleukin 1 beta
MMPs: Matrix metalloproteases
PAMPs: Pathogen-associated molecular patterns
TLR: Toll-like receptor
